# Antibiofilm Activity of Three Different Irrigation Techniques: An in Vitro Study

**DOI:** 10.3390/antibiotics8030112

**Published:** 2019-08-09

**Authors:** Caterina Eneide, Raffaella Castagnola, Cecilia Martini, Nicola Maria Grande, Francesca Bugli, Romeo Patini, Massimo Cordaro, Maurizio Sanguinetti, Giovanni Olivi, Gaetano Isola, Luca Marigo

**Affiliations:** 1 Unità Operativa Complessa (UOC) Odontoiatria Generale e Ortodonzia, Dipartimento Scienze dell’Invecchiamento, Neurologiche, Ortopediche e della Testa Collo. Fondazione Policlinico Universitario “A. Gemelli” IRCCS, 00168 Rome, Italy; 2 Istituto di Clinica Odontoiatrica, Università Cattolica del Sacro Cuore, 20123 Rome, Italy; 3 Dipartimento di Scienze di Laboratorio e Infettivologiche, Fondazione Policlinico Universitario “A. Gemelli” IRCCS, 00168 Rome, Italy; 4 Istituto di Microbiologia, Università Cattolica del Sacro Cuore, 00168 Rome, Italy; 5 Inlaser, Studio Medico Dentistico Olivi and Genovese, 00152 Rome, Italy; 6 Department of General Surgery and Surgical-Medical Specialties, Via Plebiscito 628, University of Catania, 95124 Catania, Italy

**Keywords:** biofilm, disinfection, *Enterococcus faecalis*, Root canal irrigants

## Abstract

The microbial infection of the endodontic space occurs in a necrotic tooth as a result of dental caries, trauma, periodontal disease, or previous root canal therapy. The disruption of the biofilms and the reduction of the bacterial load inside root canals are crucial for the success of root canal therapy. The aim of this study was to compare, in vitro, the antibiofilm efficacy of a novel passive sonic irrigation (PSI) device with passive ultrasonic irrigation (PUI) and conventional needle irrigation (CNI). Forty-four single-rooted human teeth were inoculated with a culture of *E. faecalis* for 28 days. The specimens were randomly divided into three groups: PUI, CNI, and PSI (*n* = 12). The activation protocols were performed using both 17% EDTA and 5.25% NaOCl. Residual bacterial biofilm was taken by means of a canal brush and colony-forming unit (CFU) were counted. The data were analyzed using one-way ANOVA and Games-Howell’s post hoc tests. A major reduction in CFU was observed in the PSI and PUI groups, in comparison with the CNI group. No difference was found (*p* > 0.05) in terms of CFU reduction between PSI and PUI. PSI could be as effective as PUI in the removal of bacterial biofilms from straight root canals.

## 1. Introduction

The bacterial infection of the pulp and root canal system occurs in a necrotic tooth as a result of dental caries, trauma, periodontal disease, or previous root canal therapy. Primary endodontic infection is the microbial invasion of necrotic pulp and plays a critical role in the development of primary apical periodontitis. Secondary endodontic infection results from a persistent intraradicular infection of teeth which have been already treated. These teeth develop secondary apical periodontitis. It is known that bacteria inside the root canal system do not live in a planktonic state, but rather in a biofilm form. The presence of a biofilm, which was detected in the apical root canals of teeth with primary and secondary apical periodontitis, has shown its important role in the apical disease process [1]. 

Consequently, lowering the bacterial load and disrupting the biofilm inside the root canal system are crucial for the success of endodontic therapy. Persistent bacteria, such as *E. faecalis*, inside the root canals, are one of the main reasons for endodontic failure [2]. Clinically, during root canal therapy, combining both mechanical instrumentation and the use of irrigant solutions have achieved a three-dimensional cleaning and disinfection of the endodontic space. Irrigation is the key step of root canal therapy. Sodium hypochlorite (NaOCl) is the most widely used irrigant solutions for root canal disinfection. This compound has demonstrated an important role in dissolving necrotic pulp tissue and debris from the root canal walls, together with its lubrication properties [3]. Conventional needle irrigation (CNI) has shown its limits in delivering NaOCl inside the root canal when complex anatomy is present. Several studies have demonstrated that the efficacy of the irrigant could be enhanced by its activation. Different activation methods have been proposed to improve the effect of irrigant solutions [4,5].

By now, the gold standard is represented by passive ultrasonic irrigation (PUI), which has shown its efficacy in both root canal disinfection and removal of the smear layer [6]. The efficacy of PUI is mainly due to cavitation and to an acoustic streaming effect. However, this technique has shown several procedural limitations, such as contact of the ultrasonic file with the dentinal wall, especially in curved root canals, which reduces its energy [7].

Regarding sonically activated irrigation, a novel sonic tip EDDY (VDW, Munich, Germany) has been introduced. This polyamide tip works at a higher frequency (6000 Hz) than other sonic devices. This passive sonic irrigation (PSI) has been demonstrated to be as effective as PUI in the removal of the smear layer from the root canal walls, but only a few studies have tested its efficacy against oral bacteria [8,9,10].

In vitro studies that have tested the antimicrobial efficacy of irrigation protocols or irrigant solutions have tried to evaluate the residual bacterial biofilm inside the root canal system [10,11]. The sampling methods may be quite different; most of the studies used a sterile paper point or direct aspiration of the canal content [9,12]. Others used dentin chips obtained by diamond bur or endodontic files that should be able to collect the biofilm [13,14]. The ideal sampling method should be simple, highly reproducible, and not prone to operator-related variation.

The aim of this in vitro study was to compare the efficacy of root canal disinfection against biofilms of *E. faecalis* using three different irrigation techniques. The null hypothesis tested was that there is no difference between the activation and no-activation groups.

## 2. Results

The results are reported in Table 1 and Table 2.

A statistically significant difference was found among groups (*p* < 0.05). There was a greater CFU reduction in groups activated sonically or ultrasonically than in the CNI group.

A significant difference was found between CNI and PUI groups (*p* = 0.008) and between CNI and PSI (*p* = 0.012). However, no difference was highlighted between the PUI and PSI groups (*p* = 0.074).

## 3. Discussion

A culture of *E. faecalis* was chosen as an infecting agent because this microorganism is commonly isolated in teeth with failed root canal treatment due to persistent chronic apical periodontitis [2,15].

*E. faecalis* can live as a planktonic cell or biofilm. The development of the biofilm takes several stages to mature to a structurally complex matrix.

The change in biofilm bacteria from sensitive to resistant against disinfecting agents occurs between two and three weeks of biofilm maturation. In our study, a four-week incubation period was performed because, at this stage, the bacterial penetration into dentinal tubules is enhanced, and a stronger biofilm is formed [16].

It has been proven that the degree of root canal disinfection is highly influenced by the size of the preparation [17]. The activation techniques of the irrigant solutions showed better results in those canals with greater preparation [18,19]. In this study, the root canals were prepared up to a final preparation size of 25.06 to test the efficacy of the activation techniques in a narrower root canal, while other similar studies have tested these techniques in samples prepared to a final size of #40 [8,20]. The activation protocols used in this study are the same as those proposed for clinical use, and the protocols specified equal the activation time and volume (in mL) of the irrigant solutions. The total activation time of 1 min was performed to enhance the kinetics of sodium hypochlorite [21].

One microbiological ex vivo study challenge is the methodology for collecting samples [6]. Most of the studies used sterile paper points or direct aspiration of canal content to collect the bacterial load inside the root canal. These methods were limited to allow the collection of only planktonic bacteria [8,11]. Therefore, the collecting method used should be able to take bacteria either in planktonic or biofilm forms to reproduce the clinical state and obtain reliable results. In this study, a thin canal brush was used to collect residual bacteria. The brush performed a mechanical action against root canal walls, disrupting the biofilm and collecting bacteria either in planktonic or biofilm form.

**Figure 1:**
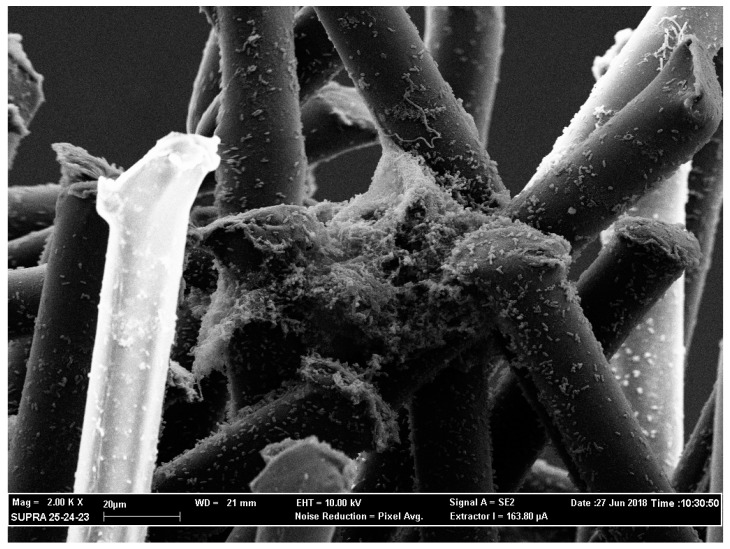
Scanning electron microscope image at 2000× magnification. The image shows the presence of the bacterial biofilm between the fibers of the canal brush.

This mechanical action was confirmed by the image obtained with a scanning electron scanning microscope (SEM) (Figure 1). Despite the simplicity of the proposed collecting method, it still remains operator-related.

Quantitative measurement of the effect of root canal disinfection protocols should be preferred over qualitative approaches. That consideration is particularly important when the overall number of the CFU in the sample is low. In this study, we used the CFU method for enumerating the residual bacteria. However, as stated from a recent revision of the United States Pharmacopeia Chapter 1223, this particular method is limited and generates approximate results. The risk of small but significant differences is high, especially when the CFU results are reported on a logarithmic scale [8,22].

A metabolic assay could be used instead of CFU for enumerating microorganisms [10]. However, this test is dependent upon the number of viable cells, so when the number of bacteria is below a specific threshold, the results should be interpreted with caution [23].

PSI produced the best numerical results. Similarly, Neuhaus et al. showed that PSI had the highest reduction of *E. faecalis* and eight other types of oral bacteria compared with PUI and CNI without a statistically significant difference [8]. In contrast, Zeng et al. found no significant difference in bacterial reduction between PSI and CNI [10]. The differences between the results presented in this study and the one reported by Zeng et al. could be related to methodological factors; Zeng et al. tested the PSI technique in root canals prepared up to a final size of 40.06, while in the current study, a preparation size of 25.06 was used. It could be speculated that in larger root canals, the “irrigation method” factor is not as significant as in smaller root canals.

Furthermore, when analyzing the percentage of intratubular bacteria-killing, Zeng et al. found that PSI was significantly more efficient than CNI in the coronal, middle, and apical portion of the canal walls, at least for the superficial layer.

In this study, no analysis of residual intratubular bacteria was performed. In addition, the use of the canal brush, as a collecting method, allows the collection of mostly biofilm that covers the root canal walls. Therefore, from the results obtained, it can be supposed that PSI is efficient in killing those bacteria layering the root canal walls. During activation, as already reported, the sonic tip quickly vaporized the irrigant solution, so it was necessary to replenish it frequently [8].

The PUI technique has been demonstrated to efficaciously reduce the bacterial load, although there were no statistically significant differences with the passive sonic irrigation technique (*p* > 0.05). During ultrasonic activation, a thin tip with a diameter #15 was used to reduce the risk of file-to-wall contact during activation [7]. Our results are in accordance with other studies, confirming the efficacy of the PUI technique, which is now considered the gold standard [24,25].

A few studies reported no differences, in terms of antimicrobial activity, between activation and no-activation protocols [10,26]. Comparing the results of this study, the activation of the irrigant solutions enhances their disinfecting ability, and the null hypothesis was rejected.

Within the limits of this in vitro study, the activation protocols showed a greater antibiofilm activity of the irrigant solutions. The conventional needle irrigation technique was significantly less effective than the other activation techniques tested. PSI proved to be at least as effective as PUI in the removal of the bacterial biofilm.

However, further studies are required to confirm these results and to test the antibiofilm activity of PSI in a complex root canal anatomy with the final aim of identifying an irrigation method that avoids the spread of bacteria beyond the apex that has recently been correlated with the onset of numerous local and systemic pathologies [27,28,29,30,31].

## 4. Materials and Methods

In total, forty-four single rooted human teeth, including both maxillary and mandibular incisors, canines and premolars, extracted for periodontal or orthodontic reasons were used. The exclusion criteria comprised those teeth with more than a single root canal, root canal treatment, internal/external resorption, immature root apices, caries, cracks, or fractures on the root surface. 

The teeth with calcifications and excessively wide canals were excluded. After extraction, the teeth were collected and stored in a saline solution to prevent drying.

A conventional access cavity was made using a diamond and tungsten carbide bur mounted on a high-speed and contra-angle handpiece with water-cooling. All root canals were scouted to the canal terminus with stainless steel k file #10 and #15 (Dentsply Maillefer, Ballagues, Switzerland). The working length was defined by subtracting 1 mm from the point at which a size #10 stainless steel k-file was visible at the apical foramen through a stereomicroscope. Then, each root canal was mechanically prepared with nickel-titanium (NiTi) rotary files Protaper Next (Dentsply Maillefer, Ballagues, Switzerland) up to size 25.06 taper.

Irrigation with 6 mL of 5.25% sodium hypochlorite Niclor 5 (OgnaLab S.r.l, Muggiò, Italy) was used throughout the mechanical instrumentation. At the end of the mechanical preparation, the canals were filled with 1 mL of 17% ethylenediaminetetraacetic acid (EDTA) solution (Coswell Group, Fumo, Italy) that was left in place for 1 min to remove the smear layer. A final sterile saline solution rinse was performed to stop the reaction of EDTA. All canals were dried using air and sterile paper points Protaper Next (Dentsply Maillefer, Ballagues, Switzerland).

The apical foramina were sealed by using a two-step bonding system iBond^®^ Total Etch (Kulzer Gmbh, Hanau, Germany) and flowable composite Filtek Supreme (3M ESPE, Seefeld, Germany). The entire root surface was coated with bonding to prevent lateral and apical leakage. The teeth were placed individually in sterilizer pouches and were autoclaved at 121 °C for 20 min. To make handling easier, the teeth were put into individual vials filled with silicone impression material Express 2 Putty (3M ESPE, Seefeld, Germany).

All the samples were sterilized using a plasma sterilizer device (Steris V-PRO^®^ maX Low-Temperature Sterilization System Group, Bordeaux, France) to avoid ruining the silicone material.

*E. faecalis* (ATCC 29212) was cultured at 37 °C in a brain heart infusion (BHI) (Difco Laboratories, Detroit, Michigan, USA) broth and agar or in BHI supplemented with 40% horse serum (BHIS) (Sigma, St. Louis, Missouri, USA). Four teeth were not infected (negative control).

Bacterial dilutions at a final concentration of McFarland 0.5 (1.5 × 10^8^ CFU/mL) were prepared in appropriate culture medium (BHI) supplemented with fungizone (16 g/mL) and used to contaminate samples. The dilutions were incubated at 37 °C for 28 days. The medium was refreshed every two days.

Four teeth were not included in the irrigation protocols (positive control).

Irrigation Protocols:

In all the tested groups, the root canals were irrigated with a total of 6 ml of 5.25% NaOCl, a total of 6 mL of 17% EDTA, and a final rinse with 2 mL of NaCl. The total activation time of 1 minute was performed with each solution. All the procedures were performed by a single operator. Each solution was refreshed between activation cycles and during every single round.

CNI (*n* = 12)

Conventional needle irrigation was performed by using a sterile syringe with a 30 gauge needle CanalPro (Coltene, Whaledent, Germany). The root canal was flushed with 6 mL of 17% EDTA and then with 6 mL of 5.25% NaOCl.

PUI *(n =* 12)

The passive ultrasonic irrigation group underwent three ultrasonic irrigation cycles of 20 s. The power was set at 20%, according to the manufacturer’s instructions. The ultrasonic tip IRRI K (VDW ULTRA, Munich, Germany) was inserted at 1 mm short of the established working length.

PSI (*n* = 12)

The sonic device EDDY (VDW, Munich, Germany) was used. This device was coupled to the airscaler SoniFlex (KaVo Dental Gmbh, Biberach, Germany) that operates at 6000 Hz. In all, three cycles of 20 s of activation were performed both with EDTA and NaOCl. The sonic tip was inserted at 1 mm short of the established working length.

Counting microorganisms

A thin canal brush Nano-Brush (Denbur, Westmont, USA) previously sterilized with a low-temperature sterilization system (Steris V-PRO^®^ maX Low-Temperature Sterilization System Group, Bordeaux, France) was inserted at 1 mm to the apex and scratched towards canal walls, for a total time of 30 s, to collect the bacteria either in planktonic or in biofilm forms. 

To confirm the mechanical action of the brush, scanning electron microscope images were taken (Figure 1). 

The brushes were then incubated in BHI broth at 37 °C under CO_2_ for 24 hours, together with a control sample, to assess sterility.

Samples were serially diluted in saline solution. In the PSI group, the CFU count of bacteria were determined without making any dilution. Aliquots of 10 μL were spread on Enterococcus-selective agar plates. After overnight incubation at 37 °C, colonies on the plates were counted to determine the number of CFU.

Data were analyzed by a one-way ANOVA and post hoc Games–Howell’s multiple comparison tests (*p* ˂ 0.05).

## 5. Conclusions

Within the limits of this in vitro study, the activation protocols showed a greater antibiofilm activity of the irrigant solutions. The conventional needle irrigation technique was significantly less effective than the other activation techniques tested. PSI proved to be at least as effective as PUI in the removal of the bacterial biofilm.

However, further studies are required to confirm these results and to test the antibiofilm activity of PSI in a complex root canal anatomy with the final aim of identifying an irrigation method that avoids the spread of bacteria beyond the apex that has recently been correlated with the onset of numerous local and systemic pathologies [27,28,29,30,31].

## Figures and Tables

**Table 1:** Mean, Median, and Standard Deviation per group of CFU after irrigation protocols.

*Experimental Groups*	Mean (SD)	Median	Range
CNI (*n* = 12)	1.42 × 10^8^ (1.1 × 10^8^)	1 × 10^8^	6 × 10^6^–3 × 10^8^
PSI (*n* = 12)	0 (0)	0	0
PUI (*n* = 12)	1.06 × 10^5^ (1.2 × 10^5^)	3.5 × 10^4^	7 × 10–3 × 10^5^

**Table 2:** CFU after irrigation protocols for each tooth.

Sample	CNI	PSI	PUI
1	1 × 10^8^	0	6 × 10^2^
2	1 × 10^8^	0	4 × 10^2^
3	1 × 10^8^	0	3 × 10^3^
4	6 × 10^6^	0	4 × 10^3^
5	4 × 10^7^	0	1 × 10^3^
6	6 × 10^7^	0	3 × 10^4^
7	4 × 10^7^	0	2 × 10^5^
8	3 × 10^8^	0	2 × 10^5^
9	3 × 10^8^	0	2 × 10^5^
10	3 × 10^8^	0	3 × 10^5^
11	3 × 10^8^	0	3 × 10^5^
12	6 × 10^7^	0	7 × 10
